# Abnormal Anterior Cingulate N-Acetylaspartate and Executive Functioning in Treatment-Resistant Depression After rTMS Therapy

**DOI:** 10.1093/ijnp/pyv059

**Published:** 2015-05-29

**Authors:** Huirong Zheng, Fujun Jia, Guangquan Guo, Dongming Quan, Gang Li, Huawang Wu, Bin Zhang, Changhe Fan, Xiajun He, Huiyan Huang

**Affiliations:** Guangdong Mental Health Center, Guangdong General Hospital, Guangdong Academy of Medical Sciences, Affiliated School of Medicine of South China University of Technology, Guangzhou, Guangdong, P.R. China (Drs Zheng, Jia, Wu, Zhang, and Fan, and Mr Guo, Quan, Li, and Ms He); Department of Radiology, Guangzhou Hui-Ai Hospital, Affiliated Brain Hospital of Guangzhou Medical University, Guangzhou, Guangdong, P.R. China (Dr Wu); Pharmacy Department of Guangdong General Hospital, Guangdong academy of medical sciences, Guangzhou, Guangdong, P.R. China (Ms Huang).

**Keywords:** 1H-MRS, anterior cingulate, depression, executive functioning, N-acetylaspartate, rTMS

## Abstract

**Background:**

Cognitive impairment is a key feature of treatment-resistant depression (TRD) and can be related to the anterior cingulate cortex (ACC) function. Repetitive transcranial magnetic stimulation (rTMS) as an antidepressant intervention has increasingly been investigated in the last two decades. However, no studies to date have investigated the association between neurobiochemical changes within the anterior cingulate and executive dysfunction measured in TRD being treated with rTMS.

**Methods:**

Thirty-two young depressed patients with treatment-resistant unipolar depression were enrolled in a double-blind, randomized study [active (n=18) vs. sham (n=14)]. ACC metabolism was investigated before and after high-frequency (15Hz) rTMS using 3-tesla proton magnetic resonance spectroscopy (1H-MRS). The results were compared with 28 age- and gender-matched healthy controls. Executive functioning was measured with the Wisconsin Card Sorting Test (WCST) among 34 subjects with TRD and 28 healthy subjects.

**Results:**

Significant reductions in N-acetylaspartate (NAA) and choline-containing

Compound levels in the left ACC were found in subjects with TRD pre-rTMS when compared with healthy controls. After successful treatment, NAA levels increased significantly in the left ACC of subjects and were not different from those of age-matched controls. In the WCST, more perseverative errors and fewer correct numbers were observed in TRD subjects at baseline. Improvements in both perseverative errors and correct numbers occurred after active rTMS. In addition, improvement of perseverative errors was positively correlated with enhancement of NAA levels in the left ACC in the active rTMS group.

**Conclusions:**

Our results suggest that the NAA concentration in the left ACC is associated with an improvement in cognitive functioning among subjects with TRD response to active rTMS.

## Introduction

Treatment-resistant depression (TRD) is a clinical condition that can present with severe and disabling symptoms, and cognitive impairment is considered to be a core deficit of refractoriness (Berlim and Turecki, 2007). TRD subjects showed worse functioning than matched healthy subjects in several cognitive domains, such as executive functioning and memory (Gutierrez et al., 2005; Richard-Devantoy et al., 2012). These deficits do not remit between episodes, and profoundly affect long-term outcomes (Richard-Devantoy et al., 2012), hence placing an enormous burden on the families of patients (Berlim and Turecki, 2007). The cognitive impairment of TRD affects millions of adults every year worldwide. Since no objective evaluation markers are available currently, diagnosis and prognosis of TRD is difficult. The pathogenesis of this disease is complicated and involves multiple biochemical, genetic, and psychological factors (Sackeim, 2001).

Functional and anatomical abnormalities of the anterior cingulate cortex (ACC) have consistently been reported in patients with major depressive disorder (Kanner, 2004; Gonul et al., 2006; Lorenzetti et al., 2009). The results of postmortem studies support the findings by showing abnormalities in the cellular structure of this region (Rajkowska, 2000). Decreased activation in the anterior cingulate gyrus has been reported in depression, and the magnitude of anterior cingulate activity has been found to predict treatment response, with lower activation during an acute episode predicting a worse response to treatment (Seminowicz et al., 2004). Treatment-related changes in the activation of different regions of the anterior cingulate gyrus have also been reported, with the most common finding being an increase in activation in the hypoactive regions during an acute depressive episode (Kennedy et al., 2001; Das et al., 2013).

Convergent findings from neuropsychological and magnetic resonance spectroscopy (MRS) studies have shown the important role of the ACC in cognition (Allman et al., 2001). For instance, the anterior cingulate gyrus is considered an executive region for emotion and cognition, essential to the modulation of executive control (Rogers et al., 2004). In addition, considering the proposed critical role of the ACC in cognition, the close functional and anatomical connectivity between the ACC and dorsolateral prefrontal cortex (DLPFC), neurochemical changes and executive control in this region in depression seem to be likely (Allman et al., 2001; Paus et al., 2001). Although abnormalities in the ACC have been observed in depression, the roles of regional metabolism remain largely unknown (Gonul et al., 2006; Drevets et al., 2008). MRS is a technique that can be used to measure the biochemical or metabolite concentrations in specific brain regions *in vivo*. Typical compounds that can be measured in proton (1H) MRS include N-acetylaspartate (NAA), glutamate (Glx), creatine (Cr), choline-containing compounds (Cho) and myo-inositol (m-Ino; Burtscher and Holtas, 2001).

NAA is a neuronal compound exclusively found in mature neurons, and therefore is thought to be a marker of neuronal integrity and viability (Benarroch, 2008). NAA is involved in the modulation of neurotransmitter release and NAA is a direct precursor for synthesis of the most concentrated neuropetide in the human brain (Moffett et al., 2007). NAA level decreases in the cingulate gyrus are associated with neuropathologies like major depressive disorder (Merkl et al., 2011) and brain ischemia (Moffett et al., 2007). However, accumulating evidence suggests that decreases in regional NAA levels are not static and may represent reversible neuronal or mitochondrial dysfunction, and that NAA decreases in primate models can be reversed after toxin withdrawal over time (Moffett et al., 2007). In line with this, Van der Hart et al. (2001) reported significantly decreased concentrations of NAA in chronically stressed animals and reversal of that by antidepressants, indicating a neurotrophic effect.

Cho is an essential precursor of acetylcholine and has been implicated in the pathophysiology of mood disorders. Choline may have a depressogenic effect on the central nervous system (Kusumakar et al., 2001). However, the precise relationship between choline signals and acetylcholine transmission in the brain is unclear. A meta-analysis of MRS study on depression showed increased Cho/Cr in the basal ganglia but no alternation of NAA (Yildiz-Yesiloglu and Ankerst, 2006). These ﬁndings mainly came from studies of major depression, and it is uncertain whether a similar pattern can be found in the refractory depressed patients (Yildiz-Yesiloglu and Ankerst, 2006; Cousins and Grunze, 2012). Since treatment-resistant depression may differ from major depression that occurs in earlier adulthood in its etiology and clinical outcome, an MRS study among TRD patients would be of interest to provide more information on these important issues.

Repetitive transcranial magnetic stimulation (rTMS) techniques have considerably advanced in the last two decades and have been increasingly applied to investigate treatments of TRD (Fitzgerald, 2003; Gaynes et al., 2014). High-frequency (5-20Hz) rTMS over the left DLPFC is usually applied for the treatment of major depression. Meta-analyses suggest rTMS can significantly improve depressive symptoms among TRD patients (Schutter, 2009). Although the neurobiological mechanisms underlying these effects are unclear (Michael et al., 2003), previous studies have indicated that reduced neurotrophic signaling may be involved in the pathophysiology of depression (Machado-Vieira et al., 2009). rTMS associated with a induced electric current may affect brain neurotrophic metabolism during neural tuning (Silvanto and Pascual-Leone, 2008; Schaller et al., 2014). Moreover, a neurotrophic mode of antidepressant action was also proposed, promoting release of nerve cells to increase postsynaptic efficacy (Hisaoka et al., 2001; Zheng et al., 2010). Several ﬁndings of functional neuroimaging studies have reported changes in regional cerebral blood flow (rCBF) and glucose metabolism in different frontal structures, including the ACC, and rCBF response in the ACC due to stimulation of the DLPFC by rTMS (Paus et al., 2001). However, it should be noted that the available data, especially on metabolism levels from depressive patients using MRS, are not consistent, and there are no studies to date on metabolic measurements within the anterior cingulate of TRD.

Although rTMS seems to be a new tool for treating TRD, reports on the executive performance of rTMS treatment in depressed patients during treatment stages are very limited (Little et al., 2000; Martis et al., 2003). The Wisconsin Card Sorting Test (WCST), a widely used neuropsychological index of prefrontal cortical function, demonstrated that depressed patients have signiﬁcant deﬁcits on multiple measures compared to healthy individuals. The WCST provided neuropsychological evidence for signiﬁcant prefrontal cortical dysfunction in depression (Merriam et al., 1999; Dunkin et al., 2000; Rogers et al., 2004). Moreover, the neuropsychological and structural-functional MRI findings appear to be consistent. The anterior cingulate is thought to be involved in the regulation of executive control and working memory (Allman et al., 2001). Cognitive impairment is a key feature of TRD and can be related to the ACC function (Rogers et al., 2004).

Whether rTMS treatment can alter neurobiochemical proﬁles of the ACC in TRD is unclear. If so, whether such concentration changes are predictors for executive functioning is also unknown. In this study, we examined cognitive deficits and altered biochemical signals in pre- and post-rTMS younger adult patients with TRD compared with healthy subjects. Our goals were to test the hypothesis that neural metabolism in the ACC may be altered in TRD by treatment with rTMS and associated with changes in executive functioning.

## Methods and Materials

### Participants and Clinical Assessments

A total of 34 patients fulfilling the diagnostic criteria for major depressive episode (DSM-IV) and referred to rTMS because of drug-treatment resistance were enrolled in this study. Twenty-eight healthy volunteer subjects (16 males, 12 female; mean ± standard deviation: 27.6±5.1 years of age) with normal neurological and psychiatric histories and examination were studied under identical conditions. All subjects were recruited by advertising or clinician referral. All the patients and the controls were right-handed.

The study involved a two-group (active and sham rTMS) randomized blinded (patients and rater) trial with MRS brain scans and clinical assessments performed prior to treatment and after 4 weeks (within 24 hours of the last rTMS session) by sequentially numbered containers. The baseline scans of two TRD patients were discarded because of motion-degraded spectral qualitys, yielding a final sample of 32 TRD patients and 28 healthy volunteers.

The patients were classified as being severely depressed based on higher Hamilton Depression Rating Scale (HAMD) scores and lengths of illness (Table 1). The diagnoses were made independently by two experienced psychiatrists, and all patients’ current episodes met the DSM-IV depressive episode criteria. The ages of the patients were from 18–40 years (mean age 26.9±5.4 years), and the mean disease course was 4.7±3.2 years. The patients underwent electroencephalographic and clinical examinations before included into the study. Exclusion criteria were: any other psychiatric axis-I or axis-II disorders, history of epileptic seizures or any other neurological disorder, any kind of metal implants, and any other clinically relevant abnormalities in their medical history or laboratory examinations. Patients with a medical history of alcohol or drug abuse were also excluded.

**Table 1.** Clinical and Demographic Characteristics of Responder and Non-Responder Patients in Active and Sham rTMS Group.

Variable	Active rTMS (n = 18)		Sham rTMS (n = 14)		
	Mean	SD	Mean	SD	*p*-value
Age (y)	26.9	6.4	26.9	4.3	0.98 ^a^
Gender (male/female; n)	12/6		9/5		0.72 ^b^
Marital status (single/married; n)	10/8		8/6		0.76 ^b^
Education (y)	12.0	2.2	12.9	2.1	0.23 ^a^
Onset age (y)	21.5	4.3	21.6	4.2	0.75 ^a^
Course (y)	4.6	3.7	4.7	2.7	0.94 ^c^
BDI					
Pre- rTMS	20.3	3.7	21.9	3.8	0.22 ^a^
Post- rTMS	11.2^***^	4.9	19.6	4.6	<0.001 ^a^
HAMD					
Pre- rTMS	23.1	3.6	23.6	3.6	0.96^a^
Post- rTMS	13.5^***^	5.1	22.9	3.4	<0.001 ^a^
PSQI					
Pre- rTMS	15.2	4.7	15.6	5.4	0.82 ^a^
Post- rTMS	10.2^***^	3.7	15.8	4.4	<0.001 ^a^
Responders	11		1		<0.001 ^d^
% improvement	48.7	18.8	23.7	10.4	<0.001 ^c^

^a^Independent sample *t*-test; ^b^Pearson χ^2^ test; ^c^Mann–Whitney U-test; ^d^Fisher^’^s exact test.

BDI, Beck Depression Inventory; HAMD, Hamilton Depression Rating Scale; PSQI, Pittsburgh sleep quality index; rTMS, repetitive transcranial magnetic stimulation; SD, standard deviation.

^***^ *p* < 0.001, significant difference from pre-rTMS

Treatment resistance was defined as failure to respond to at least two different antidepressants given for a period longer than 4 weeks at the maximum recommended dose. All patients were randomly assigned to either the active rTMS group (n = 18) or the sham rTMS group (n = 14). The sample consisted of 20 males and 12 females. There was no significant difference in relevant clinical parameters between patient groups (shown in Table 1). All patients were taking escitalopram 10mg per day for at least 2 weeks before their enrollment, and they agreed to continue the same medication during the studyI, due to the severity of illness and potential suicide risk. After a detailed description of the study, written informed consent was obtained from the subjects. The protocol was approved by the Second Xiangya Hospital of Central South University ethics committee and the studies were carried out in accordance with the Declaration of Helsinki.

Clinical symptoms were assessed by the 17-item HAMD (Hamilton, 1960), the Beck depression inventory (BDI; Beck and Beamesderfer, 1974), and the Pittsburgh Sleep Quality Index (PSQI; Buysse et al., 1989). All clinical interviews were performed by a research psychiatrist. Assessments of clinical symptoms were obtained at baseline prior to the first rTMS treatment and 4 weeks after the end of treatment. Response was defined as a HAMD reduction of 50% from baseline scores.

### Neuropsychological Assessment

The executive functions were evaluated at baseline and 4 weeks after the end of treatment using the WCST (Heaton et al., 1993). Briefly, subjects were asked to sort 48 cards on the basis of three possible categories (color, number, and shape). After six consecutive correct responses, sorting principle was changed by the test giver and subjects needed to adapt to that change. The test ended when subjects completed all six categories correctly or used all 48 cards. We selected the number of categories completed, the number of correct answers, and the number of perseverative errors as indices of executive functioning. Perseverative errors were transformed so that higher scores indicated improvement.

### rTMS Treatment

We used a figure-eight water-cooled coil (Dantec Medtronic, MagPro R30), with the extensions of the coil perpendicular to a line running from the site to the subject’s nose. The patients were instructed to be relaxed. On a separate day prior to the first treatment, the resting motor threshold (MT) for the right abductor pollicis brevis muscle was determined with an electromyograph (Shanghai, NTS-2000). Motor threshold intensity was defined as the lowest stimulation intensity that, in ten trials, induced at least five moto- evoked potentials of at least 50 uV peak-to-peak amplitude (Pascual-Leone et al., 1996; Padberg et al., 2002). The position of the left DLPFC was deﬁned as 5cm anterior to the scalp position for optimum stimulation of the right interosseus dorsalis muscle in the parasagittal plane (the thumb). This method for coil positioning has been reported to be moderately accurate in targeting DLPFC areas (George et al., 2000).

All patients were naive to rTMS prior to this study. They underwent 20 sessions of rTMS over the left DLPFC within 4 weeks, at 110% stimulation intensity related to their resting motor threshold. The center of the ﬁgure-eight coil was ﬂat over the scalp and kept at a 45° angle with the handle oriented towards the back of the head. Sham stimulation occurred in exactly the same manner as active rTMS, except that the angle of the coil, rather than being tangential to the skull, was at 90 degrees off of the skull. This produced a similar sensation on the scalp but appeared not to really stimulate the brain, since such application over the motor cortex is assumed to have little biological effects other than active condition (Lisanby et al., 2001). Other stimulation characteristics were as follows: 15 Hz, 50 trains of 4s duration, 3000 stimuli/day, 28min per session, and 20 sessions of stimulation over a 4-week period (Monday to Friday). During the first week of the treatment phase, stimulation intensity could be adjusted to 100% of MT for the sake of tolerability, but it was required to reach to 110% MT from week 2 onward.

### 1HMRSI Data Acquisition and Quantitation Protocol

**Figure 1.**
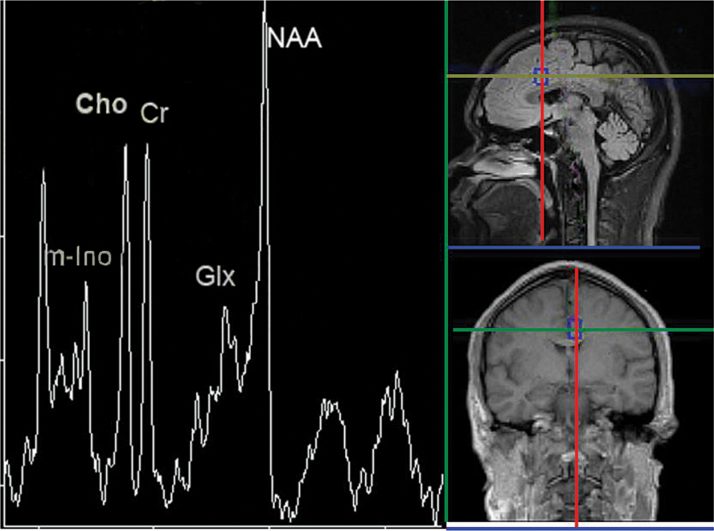
A single voxel was placed in the anterior cingulate cortex (ACC, in the BA 24) (shown on the right). This region play a prominent role in human cognitive regulation (Allman et al., 2001). A typical spectrum from this voxel is shown on the left, and demonstrates metabolite peaks for N-acetyl-aspartate (NAA), glutamate/glutamine (Glx), choline (Cho), creatine (Cr) and myo-inositol (m-Ino), which have been implicated in the pathophysiology of depression.

Magnetic Resonance Spectrum Imaging (MRSI) data was examined using a 3.0 Tesla Siemens whole body imaging system (3.0T, Trio Tim) for 32 patients (18 active rTMS, 14 sham rTMS) and 28 controls at baseline. The second MRSI scan of patients was performed at endpoint (after rTMS week 4), which was required within 24 hours after the last rTMS. The MRI protocol included T1-weighted 3D spoiled gradient echo acquisitions of transaxial and coronal orientation and T2- and fluid-attenuated inversion recovery sequence in sagittal orientation. A water-suppressed, chemical shift imaging spine echo sequence was performed using multiple voxel proton MRS. Parameters were described as follows: ﬁeld of view = 16×16cm; echo time = 30ms; repetition time = 1700ms; region of interest (ROI) = 8×8×1.5 cm3; and voxel size = 1.0×1.0×1.5cm^3^. Acquisition was repeated three times with the voxel sitting in region to reduce the errors resulted from partial volume effect and to improve signal-noise ratio. MRSI scans that covered the primary ROIs (right and left anterior cingulate, in the BA 24) were referred to readily identifiable locations on each subject’s matching high-resolution MR images (Figure 1). Head motion was minimized by comfortably securing subjects’ heads with padding within the quadrature head coil.

 A representative spectrum of a patient from the left anterior cingulate MRSI voxel is shown in Figure 1. Concentrations in proton MRS studies are expressed relative to creatine, which acts as an internal reference standard in the voxel. Each spectrum was evaluated for the peak area of NAA, m-Ino, Cho, Cr, and Glx. The ratios of m-Ino/Cr, Cho/Cr, NAA/Cr, and Glx/Cr were also calculated. The post-processing and quantitation were automatically done using the NUMARIS/4, which is a brain image software package (Siemens Syngo MR B15 version). Spectral post-processing comprised line broadening, reducing the residual water resonance, linear baseline correction, and peak integration.

### Statistical Analyses

Data was analyzed using SPSS version 18.0. Primary outcome measures were metabolites at the baseline and week 4 scans. For calculating baseline and metabolic changes after rTMS, measurements analyses of variance (ANOVA; with response as between-subject factor), *t*-tests, and Wilcoxon paired tests were performed. Demographic differences between healthy control subjects, responders, and non-responders to rTMS were calculated with Chi-square tests and *t*-tests. Secondary outcome measures were changes in the HAMD, BDI, and PSQI scores from baseline. WCST parameters between active and sham groups and responders and non-respnders were calculated with ANOVAs. Statistical inferences were made with an α threshold of *p* < 0.05 after Bonferroni correction for multiple comparisons. Finally, correlation analyses between metabolites and cognitive performance were performed with the two-tailed partial test, controlling for age, sex, and years of education.

## Results

### Clinical and Metabolic Outcome

Response was defined as a 50% reduction in the HAMD score. A total of 11 out of 18 patients responded to active rTMS treatment and 1 of 14 patients responded to sham rTMS treatment. There were no serious adverse events found in either treatment group. Statistical results of all psychopathological rating scores in the active treatment group compared to the sham group are displayed in Table 1. None of the clinical variables at baseline showed statistically significant differences between the two patient groups.

**Figure 2.**
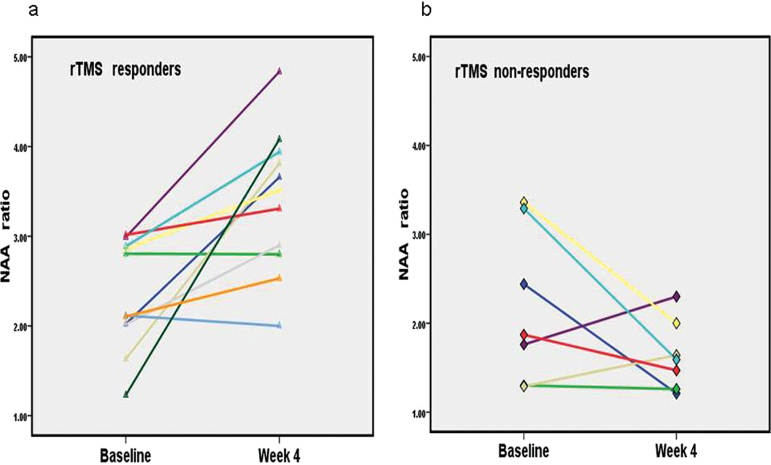
NAA levels measured by MRS (a) in treated responders (n = 11) (t = −3.69, df = 10, p = 0.004) or (b) in treated non-responders (n = 7) (t = 1.64, df = 6, p = 0.15) at baseline and after 4 weeks rTMS.

Before rTMS treatment, the concentrations of Glx, m-Ino, and Cr in patients were not statistically different from those in controls. However, patients had lower NAA and Cho ratios in the left ACC at pre-stimulation baseline levels (Table 2). After completion of 20 daily rTMS sessions, significant changes in the metabolic levels were observed in the responders to active rTMS (Table 3). NAA levels in responders (n = 11) were enhanced when compared with the pre-rTMS baseline in the left anterior cingulate (Figure 2A). After the treatment, no significant difference of the NAA ratio was observed between the responders and the controls (*t* = 0.796, df = 37, *p* = 0.43). The responders in the active group did not show changes in other MRS measures after rTMS (Table 3). In contrast, non-responding patients (n = 7) in the active group presented no signiﬁcant metabolites change in MRS measures, even though there was a tendency towards a decreased NAA level (Figure 2B).

**Table 2.** Ratios of Metabolites in the Anterior Cingulate and Results of WCST in Healthy Subjects and Patients at Baseline Between Active rTMS and Sham rTMS

Evaluation and Group	Healthy subjects	Depressive Patients		
		Active rTMS	Sham rTMS	*p*
	(n = 28)	(n = 18)	(n = 14)	
	Mean ± SD	Mean ± SD	Mean ± SD	
Measure from WCST				
Categories Completed	4.43±1.50	3.83±1.54	3.71±0.91	0.17
Perseverative Errors	8.96±4.10	13.11±6.30	12.29±3.91	0.01^a^
Number of Correct	33.43±5.38	27.56±7.86	27.72±5.31	0.01^a^
Measure from metabolites				
NAA				
Left	3.11±1.10	2.41±0.94	2.22±0.81	0.02^a^
Right	2.19±0.84	2.58±0.52	2.57±0.76	0.31
Glx				
Left	0.33±0.12	0.31±0.10	0.33±0.05	0.46
Right	1.20±0.62	1.01±0.63	1.31±0.55	0.43
Cho				
Left	0.86±0.22	0.66±0.15	0.64±0.18	0.001^a^
Right	0.83±0.20	0.77±0.21	0.83±0.22	0.39
M-Ino				
Left	0.65±0.22	0.63±0.19	0.60±0.11	0.79
Right	0.63±0.14	0.60±0.20	0.61±0.25	0.65

Cho, choline; Glx, glutamate/glutamine; m-Ino, myo-inositol; NAA, N-acetyl aspartate; rTMS, repetitive transcranial magnetic stimulation; SD, standard deviation; WCST, Wisconsin Card Sorting Test.

^a^Healthy subjects differ from active rTMS patients (Bonferroni correction)

**Table 3.** Metabolite Ratios in the Left Anterior Cingulate at Baseline and After rTMS Treatment in Responders and Non-Responders.

Metabolite ratio	Active rTMS (n = 18)		*p*	Sham rTMS (n = 14)	
	Responders	Non-responders		Responders	Non-responders
	(n = 11)	(n = 7)		(n = 1)	(n = 14)
NAA					
Pre-rTMS	2.33±0.61	2.19±0.86	0.68	1.31	2.29±0.80
Post-rTMS	3.40±0.80^*^	1.64±0.39	0.006	1.59	2.29±1.22
Glx					
Pre-rTMS	0.32±0.11	0.30±0.08	0.74	0.34	0.33±0.05
Post-rTMS	0.33±0.08	0.32±0.07	0.70	0.26	0.32±0.05
Cho					
Pre-rTMS	0.67±0.12	0.64±0.19	0.77	0.54	0.64±0.19
Post-rTMS	0.78±0.23	0.79±0.15	0.92	0.68	0.70±0.18
m-Ino					
Pre-rTMS	0.61±0.23	0.67±0.10	0.51	0.51	0.61±0.12
Post-rTMS	0.67±0.28	0.73±0.14	0.59	0.52	0.62±0.19

Only NAA was altered by successful rTMS and previously reduced m-Ino levels increased (with means and SDs).

Cho, choline-containing compounds; Glx, glutamate; m-Ino, myo-inositol; NAA, N-acetylaspartate; rTMS, repetitive transcranial magnetic stimulation; SD, standard deviation.

^*^ Signiﬁcantly different from pre- rTMS measurement (*p* < 0.01).

In addition, no significant changes of metabolic levels were observed in the 14 non-responders with sham treatment, suggesting that the metabolites are not affected by treatment in the sham group (Table 3). In the right ACC, NAA levels tended to be decreased in active responders, although this was not significant (*t* = 2.188, df = 10, *p* = 0.05). No significant effect of other metabolites, such as m-Ino, Glx, and Cho, was observed (*p* > 0.05).

### WCST Task Performance

Patients with TRD obtained significantly poorer results on the domains of perseverative errors and correct numbers when compared to healthy controls at baseline (Table 2). The patient groups did not show significant differences on the completed categories when compared to controls. Moreover, the baseline neuropsychological ratings did not differ between active and sham stimulation groups (Table 2).

**Figure 3.**
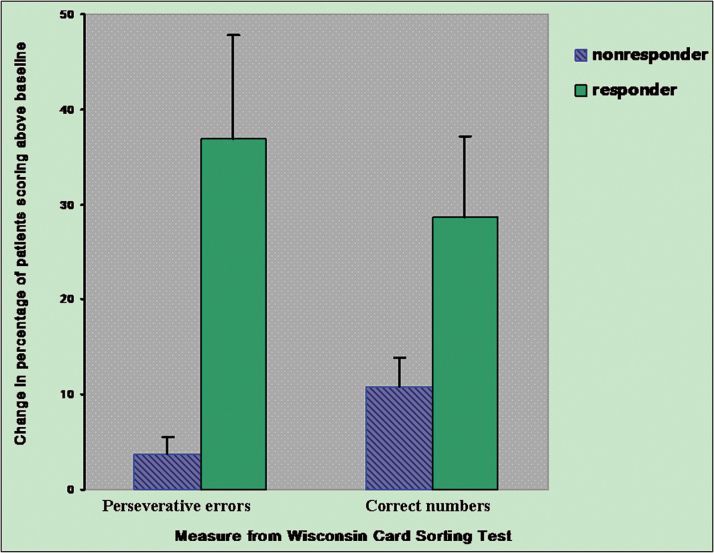
Relation of changes in executive functioning for active rTMS in responders and nonresponders.The number of perseverative errors was transformed so that higher scores indicated improvement. Responders (n = 11) showed more improvement in both perseverative errors and correct numbers, and nonresponders (n = 7) showed no noticeable improvement with statistical difference after rTMS.

After active rTMS treatment, the depressed patients in the rTMS responder group (n = 11) showed more correct numbers and fewer perseverative errors compared to the non-responders (Table 4) and performed much better compared to their baseline scores (Figure 3). Non-responding patients (n = 7) in the active group presented neither improvement of correct numbers nor significant changes of perseverative errors compared to baseline (Figure 3). In addition, the completed categories did not change during the study and did not differ between the two patient groups (Table 4). In the WCST task, no significant change on any domains of the WCST was observed in the 14 non-responders with sham treatment compared to baseline.

**Table 4.** WCST Parameters at Baseline and After rTMS Treatment in Responders and Non-Responders.

WCST parameter	All subjects	Active rTMS (n = 18)		Sham rTMS (n = 14)	
	(n = 32)	Responders	Non-responders	Responders	Non-responders
		(n = 11)	(n = 7)	(n = 1)	(n = 13)
	Mean ± SD	Mean ± SD	Mean ± SD	Mean ± SD	Mean ± SD
Categories					
Completed					
Pre-rTMS	3.75±1.31	4.09±1.70	3.43±1.27	2	3.77±0.93
Post-rTMS	4.09±1.59	4.73±1.10	4.57±0.97	3	3.38±1.07
Perseverative					
Errors					
Pre-rTMS	12.75±5.33	12.46±6.37	14.14±6.54	9	12.46±4.01
Post-rTMS	9.69±4.37^*^	7.55±3.16^*a^	15.14±3.81	10	12.54±4.79
Number of Correct					
Pre-rTMS	28.69±6.76	29.37±8.09	27.29±7.93	28	28.92±5.53
Post-rTMS	31.66±6.30^*^	34.18±4.17^*a^	30.42±4.65	29	25.54±5.34

^*^ *p* < 0.05, signiﬁcantly different from pre-rTMS measurement;

^a^ *p* < 0.05, between responders and non-responders groups (Bonferroni correction).

rTMS, repetitive transcranial magnetic stimulation; SD, standard deviation; WCST, Wisconsin Cart Sorting Test.

### Correlations of Changes in Executive Functioning and NAA Metabolic Outcome

**Figure 4.**
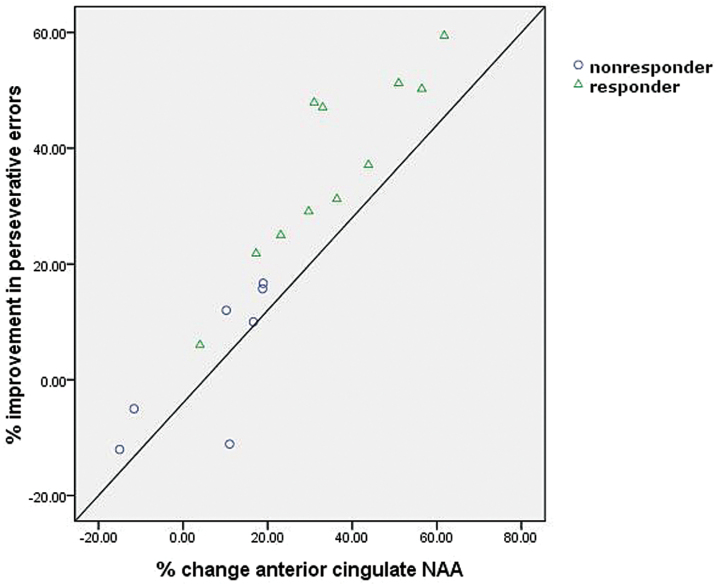
Correlation between NAA levels and change of perseverative errors in active rTMS patients (partial; N = 18, r = 0.835, p < 0.001), indicating that responder patients with higher NAA concentrations exhibited more improvement of perseverative errors.

For the NAA levels with signiﬁcant changes after rTMS, its relationship of clinical measures and WCST performance were calculated. Partial correlation analyses, controlling for sex, age, and education composition, were performed to assess associations between changes. In the WCST task, only percent improvement (i.e. decrease from baseline) of perseverative errors was positively correlated with percent change of NAA levers in left ACC in active rTMS group (n = 18, r = 0.835, *p* < 0.001; Figure 4). It revealed that responder patients with increased NAA concentrations in the left anterior cingulate exhibited more improvement of perseverative errors. No additional significant correlations for NAA changes in the left ACC were observed with gender, education, illness duration, or symptom severity.

## Discussion

This study has three major findings: (1) reduced NAA and Cho levels in the left ACC in patients with TRD compared with healthy subjects; (2) increased NAA levels in the left ACC in responders to active rTMS; and (3) a higher NAA predicts a greater improvement of executive functioning after active rTMS. We observed a NAA/Cr decrease at baseline across all patients in the left anterior cingulate gyrus gray matter, which was normalized after responding to active rTMS. The observed diminished anterior cingulate NAA concentrations in TRD are consistent with the results of previous MRS studies on depressive patients. The NAA reduction was reported in the caudate in unipolar depressed patients (Vythilingam et al., 2003) and in patients with late-life major depression (Chen et al., 2009) in the left frontal white matter. Other studies found NAA levels decreased in the ACCs of depressive patients, and the levels increased following antidepressant treatment (Gonul et al., 2006; Merkl et al., 2011). These data are consistent with our results. However, Auer et al. (2000) and Pﬂeiderer et al. (2003) did not find decreased NAA levels in depressive patients. This inconsistence is probably due to the treatment modalities, heterogeneity of depressive subtypes, and placement of voxel, and it is also possible that previous studies have been performed at lower magnetic ﬁeld strengths, most of them at 1.5T, resulting in a poor resolution spectrum. Thus, a consistent finding is yet to be identified.

We showed that active rTMS treatment increased the NAA/Cr ratio in TRD patients who responded to rTMS. It is possible that our results may be diminished neuronal viability in the anterior cingulate of depressive patients (Walter et al., 2009). Therefore, the incremental changes in NAA levels can be interpreted as an improvement of neuronal viability and integrity, which can be partly reversed by successful rTMS. Our finding supports Castrén’s (2004) work, which showed positive neurotrophic effects of antidepressants on neurons. Our functional imaging study is consistent with previous findings that the left prefrontal cortex was shown to exhibit a left-lateralized hypometabolic state with depression (Paillere Martinot et al., 2010). It has been shown that decreased metabolic activity in the ACC normalizes after symptomatic recovery with treatment (Davidson et al., 2003; Mayberg, 2003). Our finding of improvement in neuronal viability in the left ACC with active rTMS treatment supports these previous functional studies.

There was a tendency towards a decreased NAA level in nonresponders, which indicates that a neurotrophic effect might be important for rTMS efficacy. Differences between responders and nonresponders to rTMS were observed by Richieri et al. (2011) using single-photon emission computerized tomography. They found that cerebral blood flow is reduced pre-rTMS and increased after rTMS only in the responders. The most robust changes were observed in the anterior cingulate, which is part of the medial frontal cortex. However, the relationship between the metabolic activity of neurons and NAA levels remained unclear in that study. On the other hand, a drop in NAA with non-responders who got active rTMS also potentially indicates that TRD subjects whose symptoms weren’t in remission to active rTMS demonstrated further decompensation of metabolism.

Our result that Cho is substantially decreased in the left ACC suggests a local disturbance of cellular membrane metabolism (Paillere Martinot et al., 2010), of glial cell density (Gupta et al., 2000), or both, and may represent a 1H-MRS metabolite correlation of the metabolic and volumetric abnormalities seen in previous PET (Drevets et al., 2008). The presence of multiple findings such as these may implicate a variety of neural circuits in the emotional manifestations of TRD. In patients with mood disorders, glial and neuronal deficits localized to subsections of the anterior cingulate and prefrontal cortices have been reported (Rajkowska, 2000; Cotter et al., 2001). On the other hand, the absence of changes in Cho/Cr levers in responders might indicate that reduced membrane turnover in the anterior cingulate is caused not by state-dependent alterations but by a predisposing factor for depressive disorder (Yildiz-Yesiloglu and Ankerst, 2006).

In accordance with previous neuropsychological and functional imaging findings, executive dysfunction seems to be particularly prominent in patients with depression (Murrough et al., 2011). Consistent with the neurochemical reduction in the left ACC, our study showed that TRD subjects are impaired in performance on executive functions compared with healthy controls. Specifically, TRD subjects had a decline in correct numbers and an incremental change in perseverative errors of the WCST compared with control subjects. Our results indicate that the patients do not deteriorate in executive performance during the study, and even improve, with higher numbers of correct choices and fewer perseverative errors on the WCST in the active group of responders. Some studies have found that TRD patients showed significant improvements in response speed, procedural learning, and verbal and visuospatial memory in cognitive examinations after being treated with rTMS (Martis et al., 2003; Schulze-Rauschenbach et al., 2005). Although some antidepressants appear to improve cognition in affected individuals, some impairments appear to be resistant to treatment, therefore warranting the development of targeted treatment strategies to improve cognitive functioning directly (Trivedi and Greer, 2014). Furthermore, an association was found between neurochemical differences in patients with TRD and variables measuring executive skills in the present study: the increase of NAA levels in the left ACC were associated with better performance during the WCST in responders. There is not the overlap between symptomatic examinations and metabolic abnormalities which was found in the cingulate cortex by successful rTMS, suggesting that this area might majorly account for the presence of cognitive impairment (Wang et al., 2008) or that rTMS affects the complex neural metabolite in a different way from traditional medication (Zheng et al., 2010). These findings may also highlight the importance of considering metabolic profiles together in understanding the effects of rTMS. Furthermore, NAA alterations in the left ACC were not significantly related to clinical symptom measures, possibly indicating a neural dissociation between depressive psychopathology and cognitive dysfunction (Vasic et al., 2008). Some studies seem to support the idea that the ACC abnormalities in depression are related to cognitive deficits (Allman et al., 2001), but neurons and glial cells may respond differently during cognitive tasks (Burghardt et al., 2012), which may be related to the inconsistency of ﬁndings across neuroimaging and cognitive challenge studies. To our knowledge, this study is the first to show that NAA levels in the ACC are positively correlated with executive functioning improvement in TRD by rTMS treatment.

There are some limitations in this study. First, these results need a larger patient population and rTMS treaters were not blinded. For example, the small sub-sample sizes may compromise the power to detect significant differences in the prefrontal neurochemistry at endpoint. There was little effect on rTMS treaters, since we had standard training before experiment to reduce the influence. Second, our participants received continuing drug treatment, which might alter brain metabolism (Kennedy et al., 2001; Baeken et al., 2009). However, the effect of this confounding was minimized by restriction to a single drug and by ensuring stable doses for at least 2 weeks before inclusion. Third, practice effects might explain a part of the variance of WCST. However, since all patients took the tests at both timepoints (baseline and endpoint), it is unlikely that the practice effects solely explain these observations. Finally, it might be better to recruit a treatment-responsive depression comparison group and apply a neuronavigational procedure to assess rTMS targeting in a further longitudinal study.

In conclusion, this is the first pilot study of metabolite measurement *in vivo* linked to the executive dysfunction of TRD treated with rTMS. Specifically, elevated NAA levels in the left ACC of patients who responded to active rTMS are associated with ameliorated cognitive performance. This result supports a putative cognitive role in the ACC. This study also indicates that examination of metabolite and cognitive profiles may provide a new approach to understand the novel antidepressant exploration of rTMS.

## Statement of Interest

All authors report no biomedical ﬁnancial interests or potential conﬂicts of interest.
